# Supplementary material for: Chromatin heterogeneity modulates nuclear condensate dynamics and phase behavior
Source: Nat Commun. 2025 Jul 11;16:6406. doi: 10.1038/s41467-025-60771-9 (PMC12254321; doi:10.1038/s41467-025-60771-9)
Supplement: Supplementary file 1 — Supplementary Information [file 41467_2025_60771_MOESM1_ESM.pdf]

Supplementary Information

Chromatin Heterogeneity Modulates Nuclear Condensate Dynamics and Phase Behavior

Jing Xia[1], Jessica Z. Zhao[1], Amy R. Strom[1], Clifford P. Brangwynne*[1,2,3,4]

1. Department of Chemical and Biological Engineering, Princeton University, Princeton, NJ, USA
2. Omenn-Darling Bioengineering Institute, Princeton University, Princeton, NJ, USA
3. Princeton Materials Institute, Princeton University, Princeton, NJ, USA
4. Howard Hughes Medical Institute, Princeton University, Princeton, NJ, USA

*Correspondence: cbrangwy@princeton.edu

Supplementary Discussion

Side effects of drug treatment

When cells are treated with DZNep or TSA, we observe slight toxicity that affects a small subset of cells, as indicated by the slight decrease in relative cell number after 32 hours (**Supplementary Fig. 21**). To ensure accurate analysis, we exclude these unhealthy cells from our analysis by examining cell morphology for signs of shrinkage, bleb formation, or irregular nuclear morphology. The most significant changes occur during mitosis, where chromatin forms highly compacted chromosomes. These mitotic cells are excluded from our analysis due to their distinct chromatin organization, allowing us to focus solely on interphase cells. Although drug treatment causes a slight redistribution of cells across different interphase stages (**Supplementary Fig. 4(c)**), we do not observe significant differences in chromatin heterogeneity among these stages (**Supplementary Fig. 22**), and therefore do not expect this to significantly impact our system.

Supplementary figures

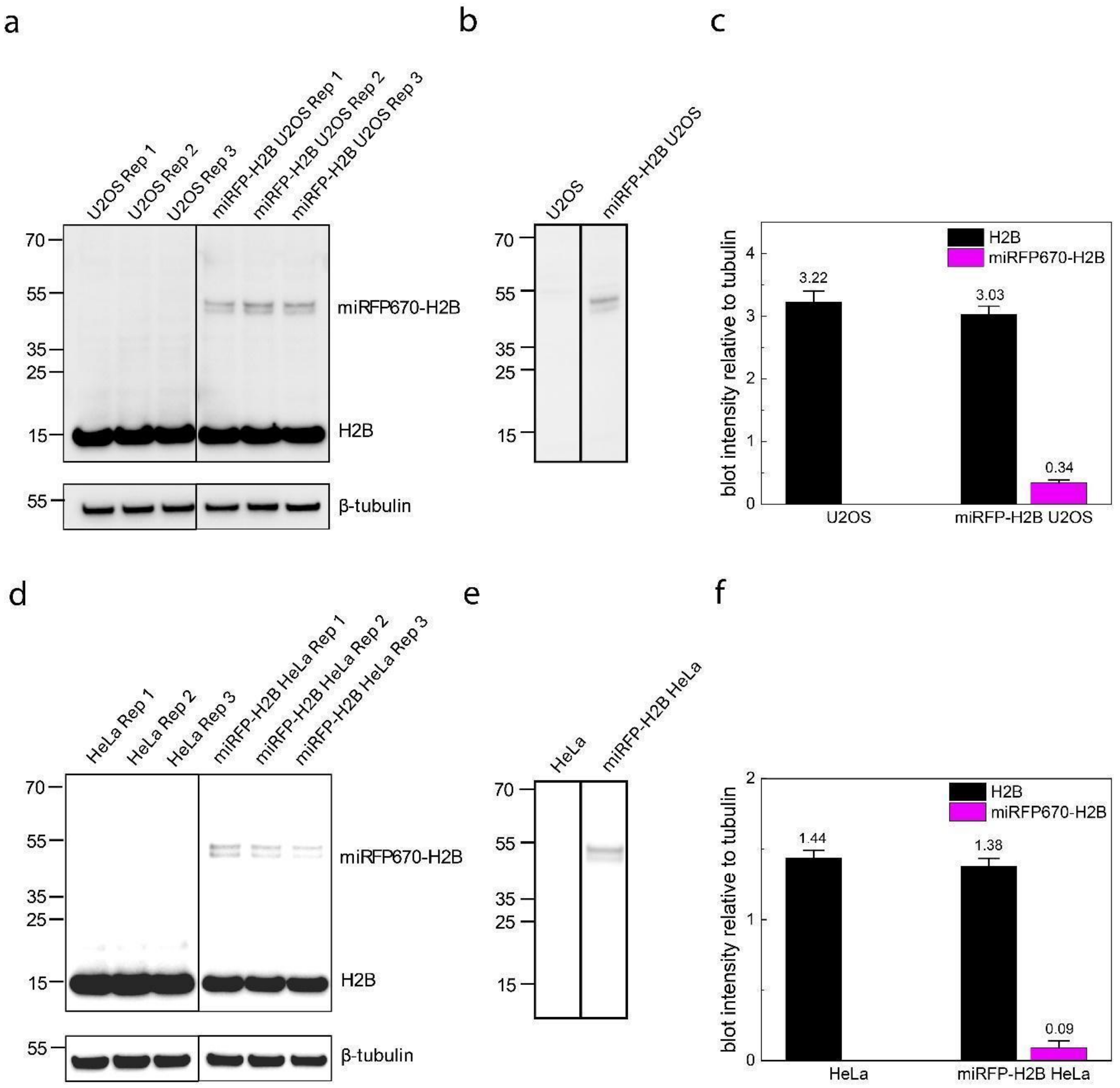

Supplementary Fig. 1. Western blot of H2B in wild-type and miRFP670-H2B transduced cells
(a), Western blot of wild-type and miRFP670-H2B U2OS cells, stained with anti-H2B antibody. β-Tubulin is used as loading control and stained with anti-β-tubulin antibody.
(b), Western blot of wild-type and miRFP670-H2B U2OS cells, stained with anti-miRFP antibody.
(c), Quantification of the band intensity of western blot image of U2OS cells shown in (a), normalized by tubulin loading control. Data is averaged from three biological replicates. Data is represented as Mean ± STD.
(d), Western blot of wild-type and miRFP670-H2B HeLa cells, stained with anti-H2B antibody. β-Tubulin is used as loading control and stained with anti- β-tubulin antibody.
(e), Western blot of wild-type and miRFP670-H2B HeLa cells, stained with anti-miRFP antibody.

(f), Quantification of the band intensity of western blot image of HeLa cells shown in (d), normalized by tubulin loading control. Data is averaged from three biological replicates. Data is represented as Mean ± STD.

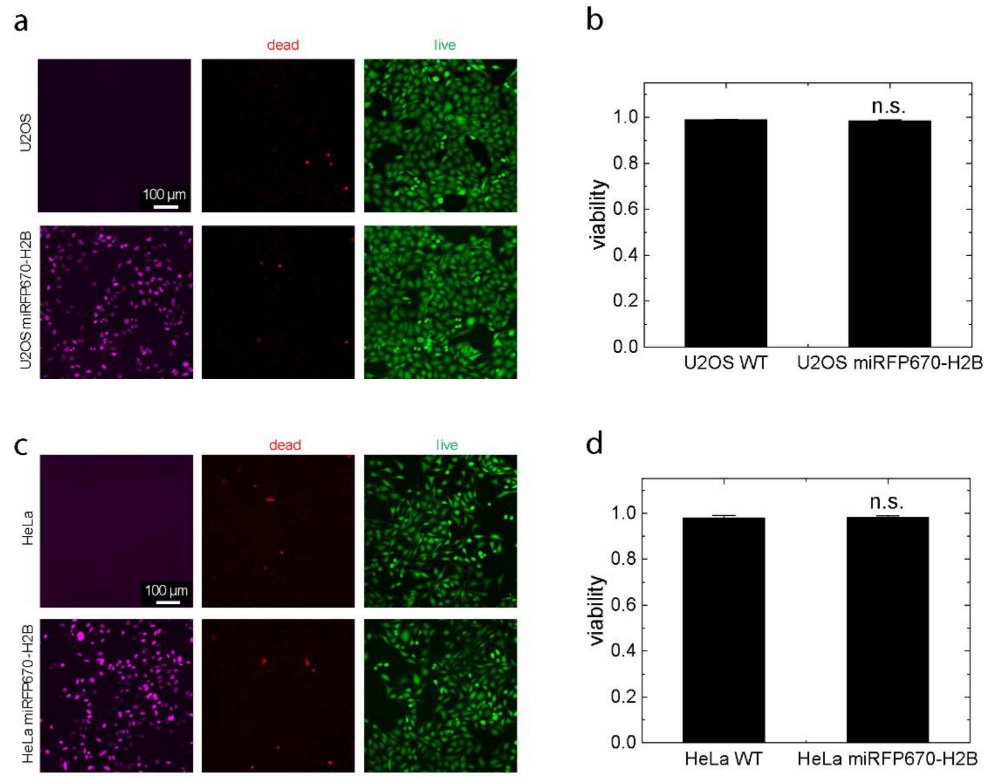

Supplementary Fig. 2. Quantification of the viability of wild-type and miRFP670-H2B transduced cells

(a), Confocal images of viability assay of the wild-type and miRFP670-H2B U2OS cells. Live cells are stained green and dead cells are stained red. Data is averaged from four biological replicates. Data is represented as Mean ± STD.

(b), Quantification of the viability of wild-type and miRFP670-H2B transduced U2OS cells. Data is averaged from four biological replicates. Data is represented as Mean ± STD. n.s., no statistical significance is found according to the t-test.

(c), Confocal images of viability assay of the wild-type and miRFP670-H2B HeLa cells. Live cells are stained green and dead cells are stained red. Data is averaged from four biological replicates. Data is represented as Mean ± STD.

(d), Quantification of the viability of wild-type and miRFP670-H2B transduced HeLa cells. Data is averaged from four biological replicates. Data is represented as Mean ± STD. n.s., no statistical significance is found according to the t-test.

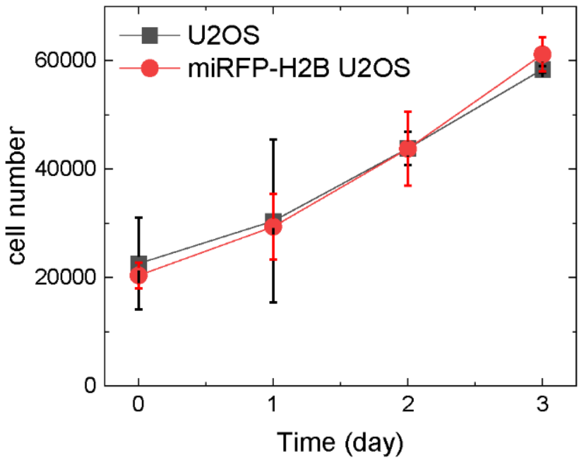

Supplementary Fig. 3. The proliferation of wild-type and miRFP670-H2B transduced U2OS cells
Cell numbers of wild-type and miRFP670-H2B cells at various culture days were quantified using the CCK8 cell counting assay. Data are averaged from more than two biological replicates and presented as Mean ± STD.

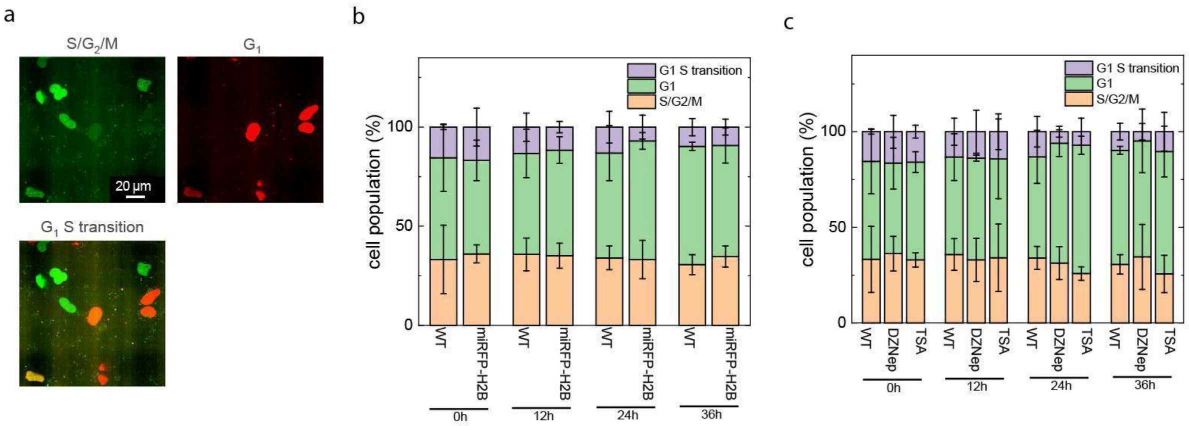

Supplementary Fig. 4. Cell cycle distribution of wild-type and miRFP670-H2B transduced U2OS cells
(a), Confocal images of wild-type cells with transduced Fluorescent Ubiquitination-based Cell Cycle Indicator (FUCCI). Cells in the S/G2/M phase are expressing monomeric Azami Green reporter only, and cells in the G1 phase are expressing monomeric Kusabira Orange 2 reporter (mKO2) only. Cells in the transition from the G1 phase to S phase are expressing both mAZ and mKO2.
(b), Quantification of cell cycle distribution in wild-type and miRFP670-H2B-transduced cells, both of which are transduced with the FUCCI indicator. Cells were quantified every 12 hours over a 36-hour period. Data are averaged from four biological replicates.
(c), Quantification of cell cycle distribution in cells with no drug, DZNep and TSA treatment. Cells were quantified every 12 hours over a 36-hour period. Data are averaged from four biological replicates.

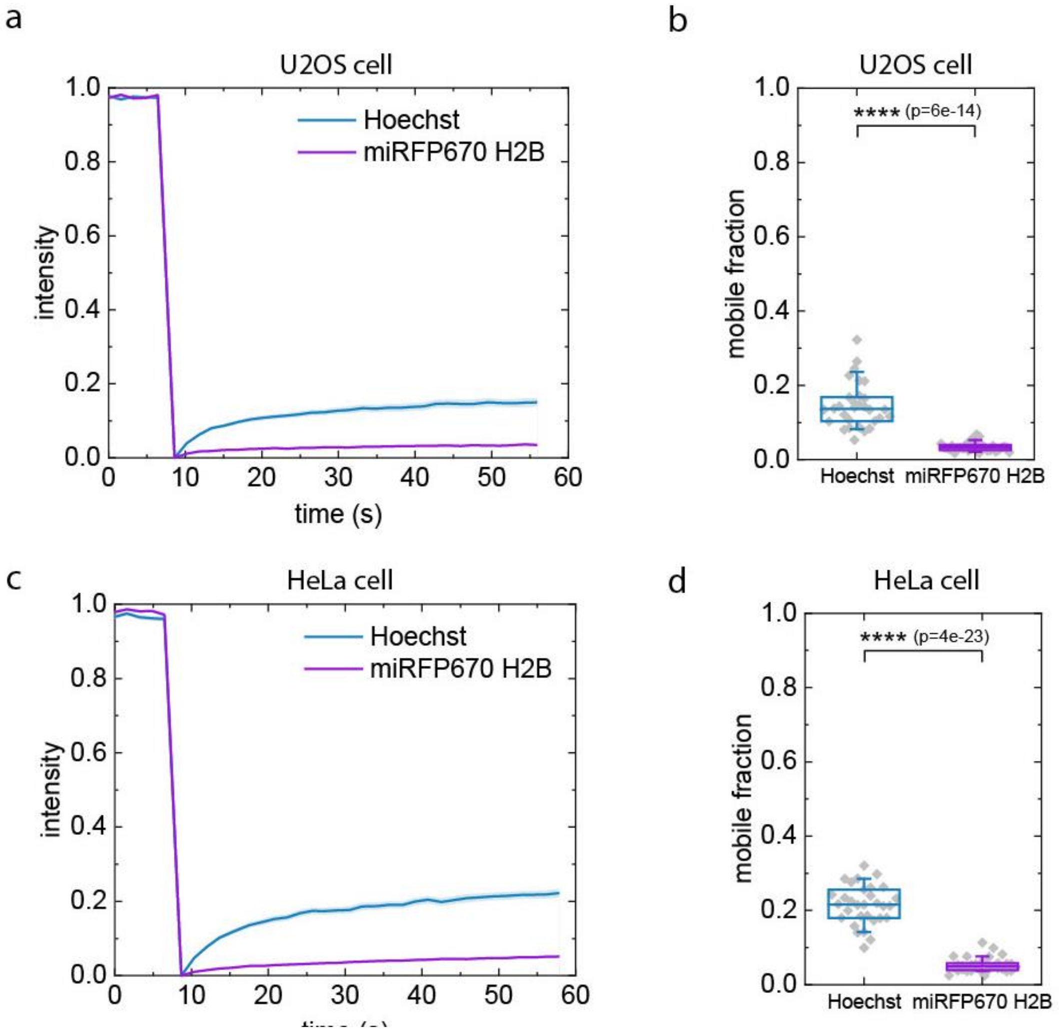

Supplementary Fig. 5. Fluorescence recovery after photobleaching (FRAP) of the miRFP670-H2B and DNA (Hoechst stain) of cells

(a), Fluorescence recovery after photobleaching (FRAP) curves of the two components of the chromatin, miRFP670-H2B and DNA(stained by Hoechst) in U2OS cells. Data is represented as Mean ± SEM. n = 30 nuclei from three biological replicates.

(b), Mobile fraction of the fluorescence recovery of Hoechst and miRFP-H2B in U2OS cell nuclei, data is analyzed by fitting the recovery curve using a single exponential recovery function. n = 30 nuclei from three biological replicates. ****, $p < 0.0001$ according to t-test.

(c), Fluorescence recovery after photobleaching (FRAP) curves of the two components of the chromatin, miRFP670-H2B and DNA(stained by Hoechst) in HeLa cells. Data is represented as Mean ± SEM. n = 31 nuclei from three biological replicates.

(d). Mobile fraction of the fluorescence recovery of Hoechst and miRFP-H2B in HeLa cell nuclei, data is analyzed by fitting the recovery curve using a single exponential recovery function. Measured from n = 31 nuclei from three biological replicates. ****, $p < 0.0001$ according to t-test.

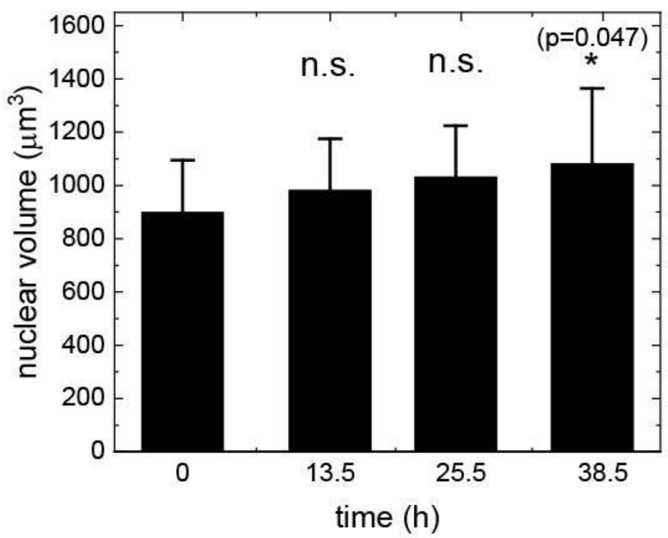

Supplementary Fig. 6. Nuclear volume of U2OS cells treated with drugs

Quantification of nuclear volume of cells treated with TSA for 0h, 13.5h, 25.5h and 38.5h. n = 17 nuclei from two biological replicates are used for statistical analysis. n.s., no statistical significance is found; *, $p < 0.05$ according to t-test.

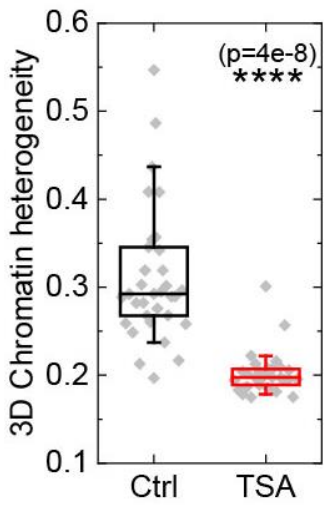

Supplementary Fig. 7. Quantification of chromatin heterogeneity in 3D of U2OS cell before and after TSA treatment

Chromatin heterogeneity of cells before and after TSA treatment. The measurements are performed on approximately 4 μm-thick 3D section of the nucleus. n=34, 29 nuclei, respectively, from three biological replicates are used for statistical analysis. ****, $p < 0.0001$ according to t-test.

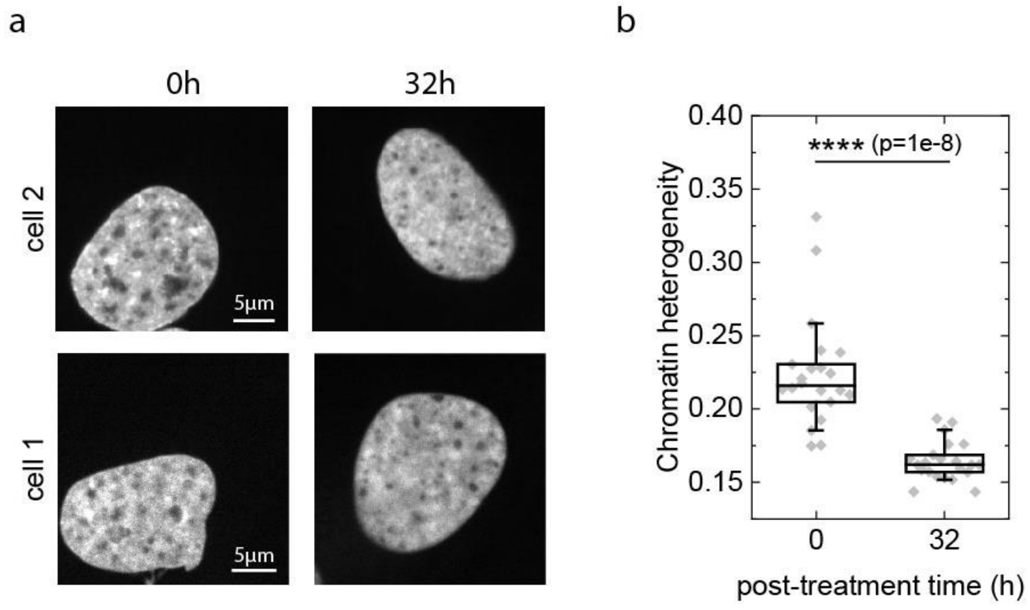

Supplementary Fig. 8. Quantification of chromatin heterogeneity in HeLa cell with TSA treatment

(a), Representative images of miRFP670-H2B tagged nuclei of the same cell before and after 32h TSA treatment.

(b), Nuclear heterogeneity of cells before and after TSA treatment (32h), calculated as the standard deviation divided by the mean of the intensity with background intensity subtracted, n=22 nuclei from three replicates are used for statistical analysis. t-tests are performed to compare the chromatin heterogeneity. ****, $p < 0.0001$ according to t-test.

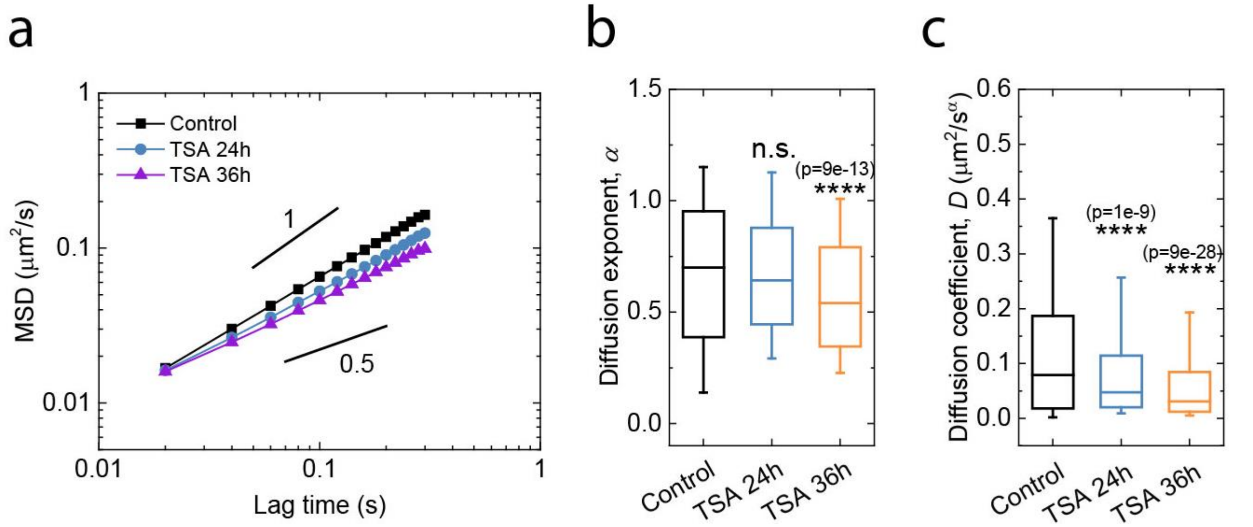

Supplementary Fig. 9. GEM40 dynamics in U2OS cells before and after TSA treatment.

(a), MSD of GEM40 in nuclei of cells under TSA treatment for 0h (n=38), 24h (n=35), and 36h (n=40). Black solid lines are visual guides for slopes of 0.5 and 1, respectively. Three biological replicates are used to calculate the mean of MSD.

(b) and (c), Diffusive exponent and diffusion coefficient of GEM40 in nuclei of cells under TSA treatment of 0h (n=38), 24h (n=35), and 36h (n=40. Three biological replicates are used for statistical analysis. n.s., non-significant ; ****, $p < 0.0001$ according to t-test.

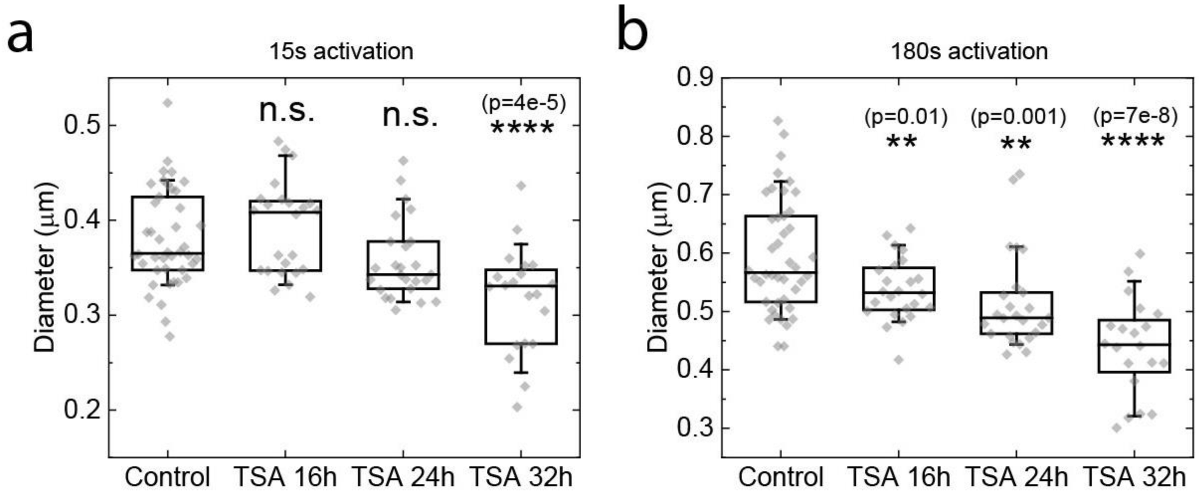

Supplementary Fig. 10. Quantification of Corelet condensates size in U2OS cells treated with TSA
(a), Averaged condensate diameter per U2OS cell nuclei subjected to TSA treatment for 16h (n=24), 24h (n=25), 32h (n=18) and those untreated (n=38), both illuminated with blue light for 15 seconds. Data is represented as three biological replicates. n.s., non-significant; ****, p < 0.0001 according to t-test.
(b), Averaged condensate diameter per U2OS cell nuclei subjected to TSA treatmentfor 16h (n=24), 24h (n=25), 32h (n=18) and those untreated (n=38), both illuminated with blue light for 180 seconds. Data is represented as three biological replicates. **, p < 0.01 according to t-test; ****, p < 0.0001 according to t-test.

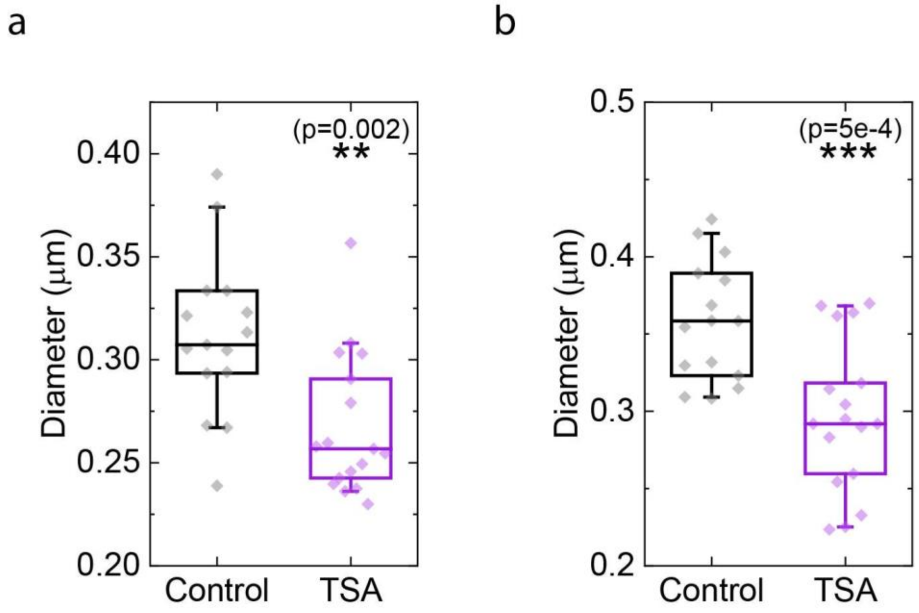

Supplementary Fig. 11. Quantification of Corelet condensates size in HeLa cells treated with TSA

(a), Averaged condensate diameter per HeLa cell nuclei subjected to TSA treatment for 26h (n=18) and those untreated (n=15), both illuminated with blue light for 15 seconds. Data is represented as three biological replicates. **, p < 0.01 according to t-test.
(b), Averaged condensate diameter per HeLa cell nuclei subjected to TSA treatment for 26h (n=18) and those untreated (n=15), both illuminated with blue light for 90 seconds. Data is represented as three biological replicates. ***, p < 0.001 according to t-test.

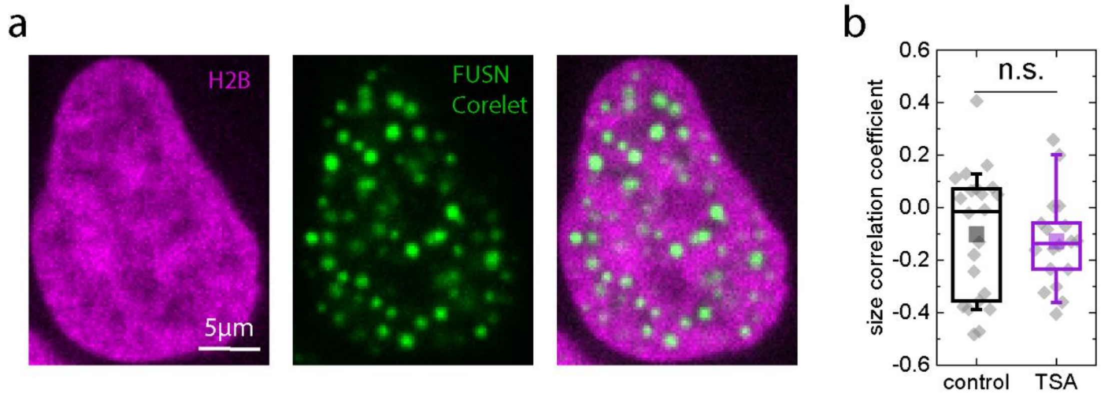

Supplementary Fig. 12. Correlation of H2B intensity and condensates sizes in U2OS cells
(a), Representative confocal images of FUSN IDR Corelets in a cell nucleus co-expressing miRFP670-H2B.
(b), Correlation coefficient of condensate size with the local H2B intensity in the area occupied by the condensate per cell nuclei subjected to TSA treatment (n=18) and those untreated (n=22). Two biological replicates are used for statistics.

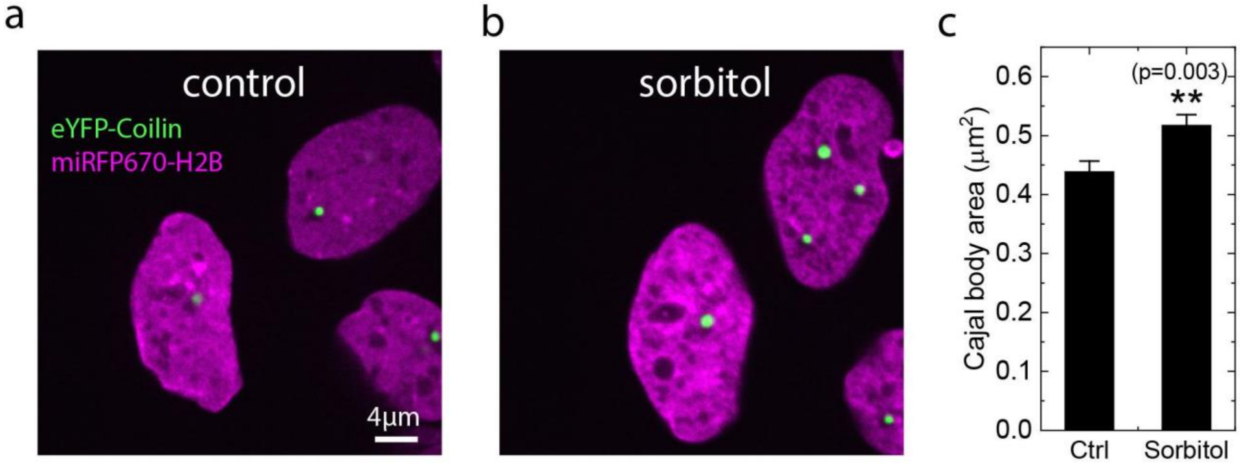

Supplementary Fig. 13. Increased chromatin heterogeneity in U2OS cell nuclei following sorbitol treatment increases the size of the Cajal body.
(a) and (b), Confocal fluorescence images of nuclei labeled with miRFP-670 and Cajal bodies tagged with eYFP-Coilin. Cells were treated without (a) and with (b) 4% sorbitol for 0.5 hours.

(c), Quantification of the mean area of Cajal body per U2OS cell without and with sorbitol treatment. n=300 U2OS cell nuclei from 3 biological replicates are used for statistics.

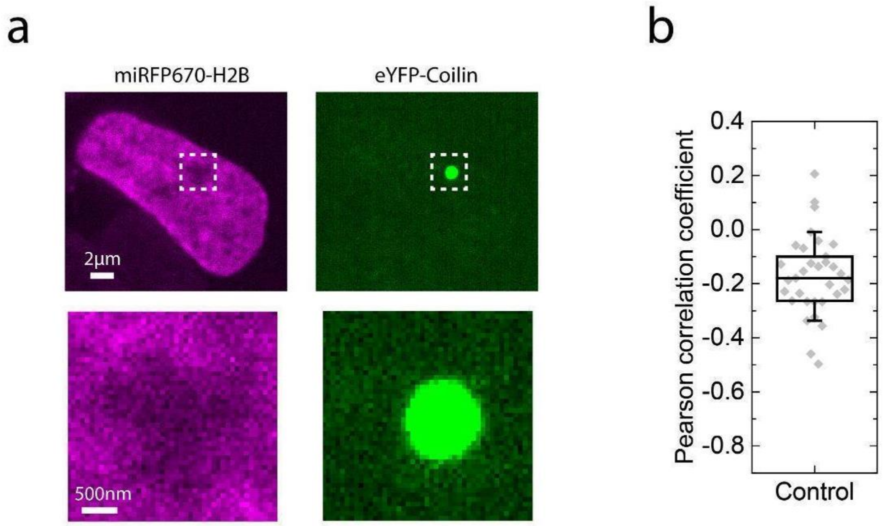

Supplementary Fig. 14. Cajal bodies are excluded from the chromatin network of U2OS cells
(a), Confocal fluorescence images show the nucleus labeled with miRFP-670 and the Cajal body tagged with eYFP-Coilin within cells. The bottom row presents zoomed-in figures for enhanced detail.
(b), Pearson correlation coefficient of the fluorescence intensity of eYFP-Coilin and miRFP670-H2B (n=33). Two biological replicates are used for statistics.

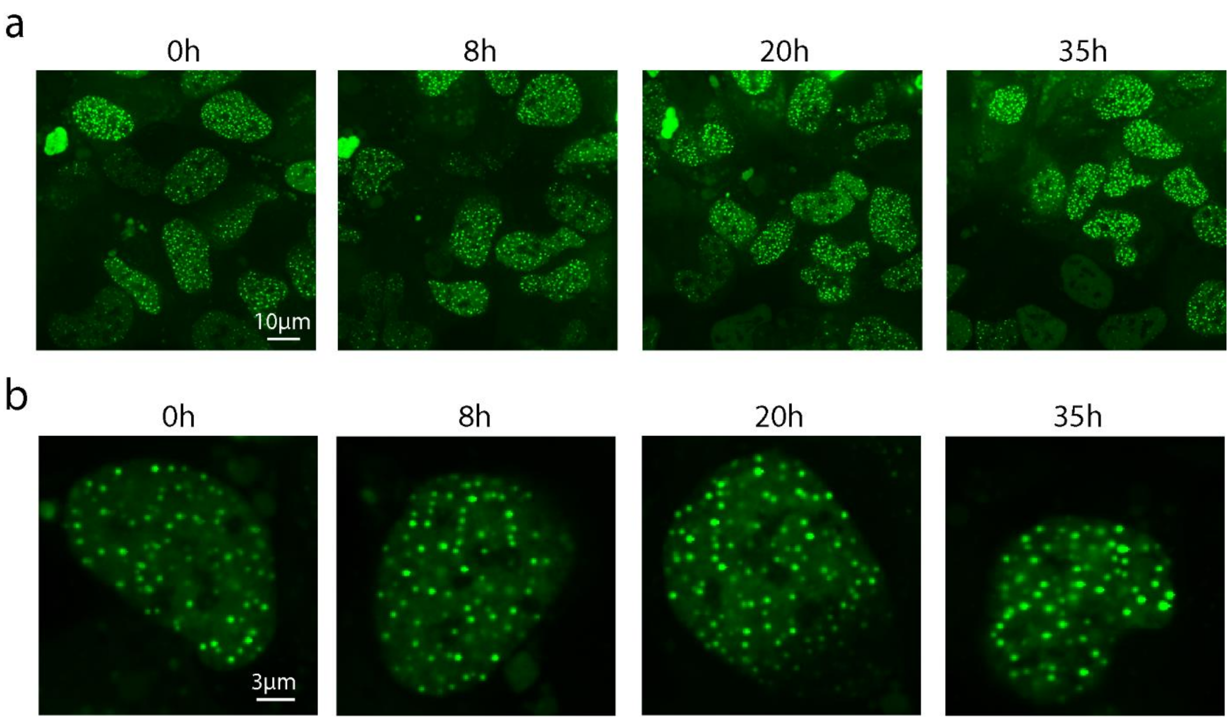

Supplementary Fig. 15. No observable inhibition of phase separation in U2OS cells before and after adding DMSO for 32h

(a), Fluorescence images show the formation of light-inducible condensates within the same field of view after blue light activation. These cells have been treated with DMSO for durations of 0h, 8h, 20h, and 35h. The experiment was repeated three times independently with similar results.

(b), Fluorescence images show the formation of light-inducible condensates within the same cell after blue light activation. The representative cell has been tracked and treated with DMSO for durations of 0h, 8h, 20h, and 35h. The experiment was repeated three times independently with similar results.

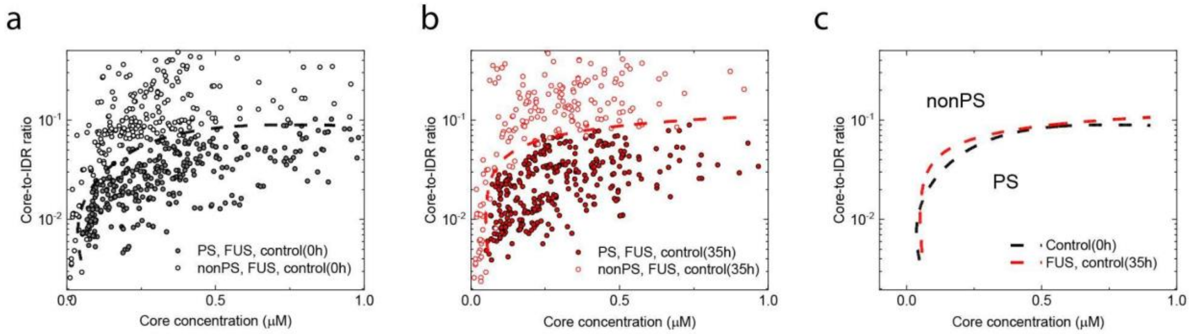

Supplementary Fig. 16. No shift of phase boundary in U2OS cells before and after adding DMSO for 32h

(a) and (b), Phase diagrams depict the boundary between phase-separated (PS) cells and non-phase-separated (nonPS) cells expressing FUS_N IDR Corelets. The axes represent Core concentration and Core-to-IDR ratio. These diagrams correspond to cells before (a) (n=594) and 32h after adding DMSO (b) (n=521). The data is represented as three biological replicates

(c), Comparison of phase boundary of photo-activated FUS$_N$ IDR Corelets in cells before(a) and 32h(b) after adding DMSO.

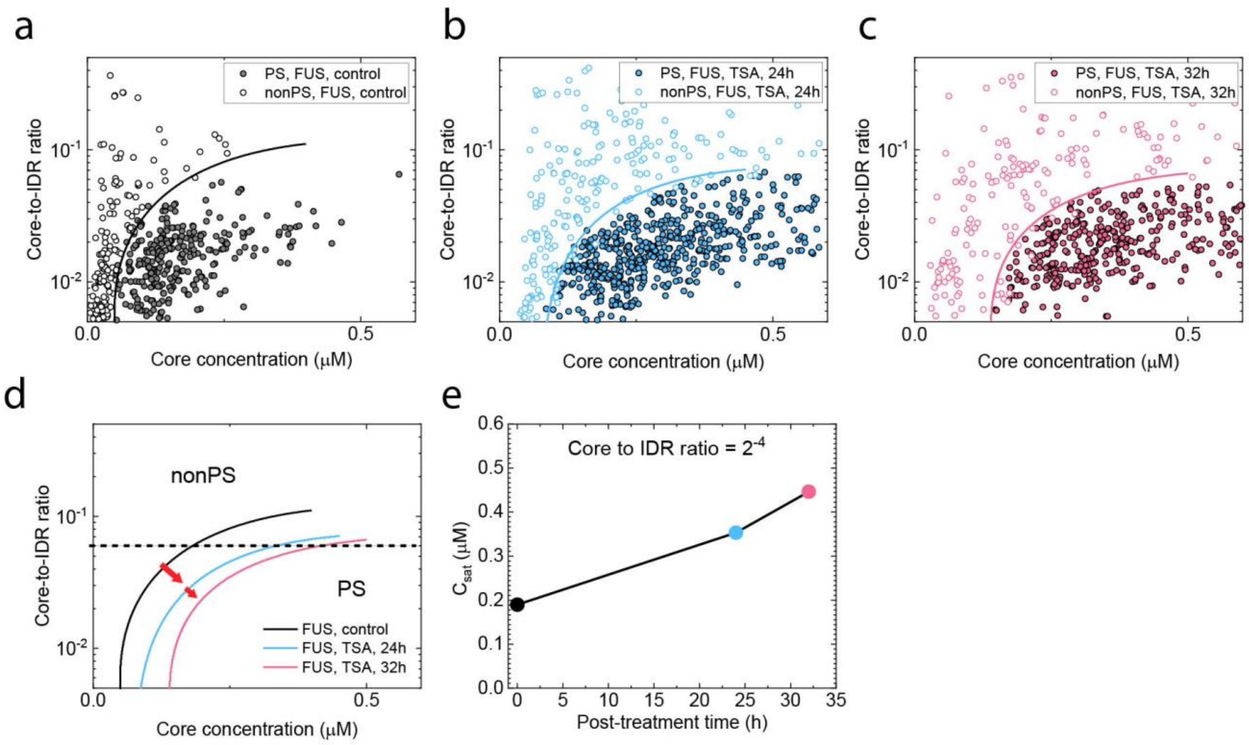

Supplementary Fig. 17. Inhibition of phase separation and change in phase diagram due to homogenization of chromatin network in HeLa cells

(a), (b) and (c), Phase diagrams depict the boundary between phase-separated (PS) cells and non-phase-separated (nonPS) cells expressing FUS$_N$ IDR Corelets. The axes represent Core concentration and Core-to-IDR ratio. These diagrams correspond to cells subjected to TSA treatment for durations of 0h (a) (n=466), 24h (b) (n=891), and 32h (c) (n=645). The data is represented as three biological replicates.

(d), Shift of phase diagram of photo-activated FUS$_N$ IDR Corelets in cells following TSA treatment of 0h(a), 24h(b), and 32h(c).

(e), Change in the core concentration at the phase boundary for a constant Core to IDR ratio of 1/16, as a function of TSA treatment duration. Data are quantified from (d).

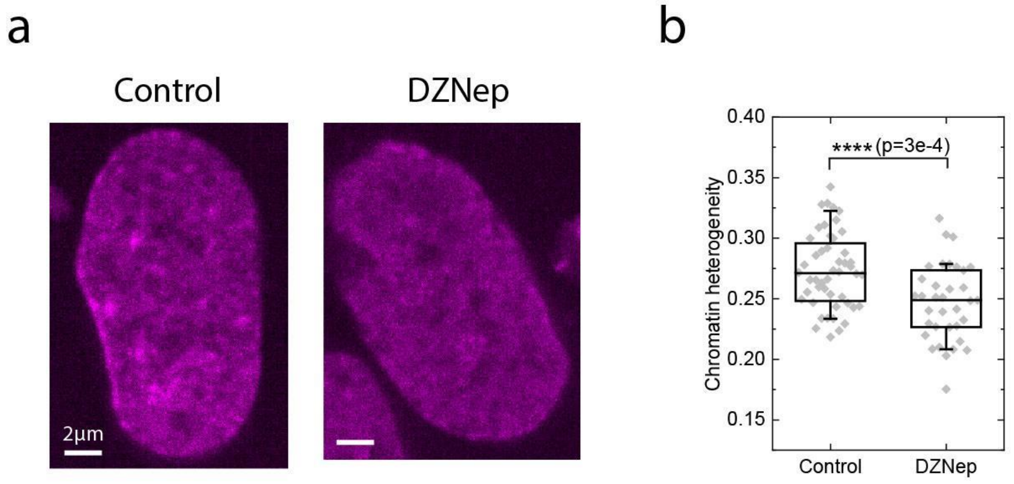

Supplementary Fig. 18. Decrease of chromatin heterogeneity in nuclei of U2OS cells under DZNep treatment

(a), Confocal fluorescence Images showing miRFP-670 tagged nuclei of cells with and without DZNep treatment. (b), Chromatin heterogeneity of cells with (n=35) and without (n=48) DZNep treatment, calculated as the standard deviation divided by the mean of the intensity with background intensity subtracted. Two biological replicates are used for statistics.

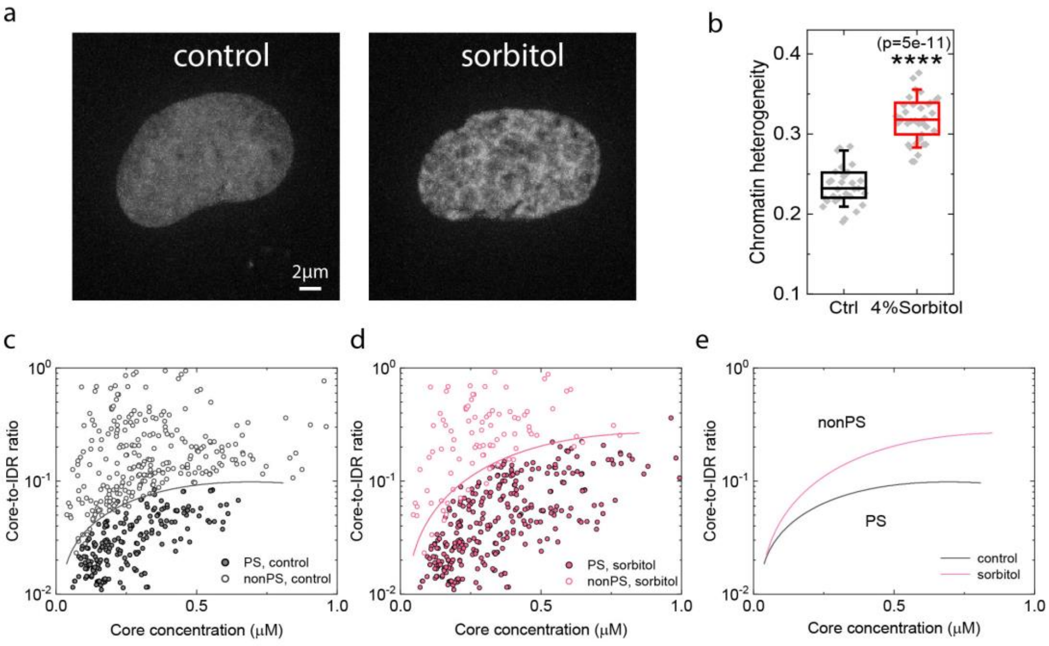

Supplementary Fig. 19. An increase in chromatin heterogeneity in U2OS cell nuclei under sorbitol treatment promotes phase separation and alters the phase diagram

(a), Confocal fluorescence Images showing miRFP-670 tagged nuclei of cells without and with (0.5h) sorbitol treatment.

(b), Chromatin heterogeneity of cells with (n=39) and without (n=33) sorbitol treatment , calculated as the standard deviation divided by the mean of the intensity with background intensity subtracted. Three biological replicates are used for statistics.
(c) and (d), Phase diagrams depict the boundary between phase-separated (PS) cells and non-phase-separated (nonPS) cells expressing FUS_N IDR Corelets. The axes represent Core concentration and Core-to-IDR ratio. These diagrams correspond to cells subjected to sorbitol treatment for durations of 0h (c) (n=620), and 0.5h (d) (n=521). The data is represented as three biological replicates.
(e), Shift of phase diagram of photo-activated FUS_N IDR Corelets in cells following sorbitol treatment of 0h(c), and 0.5h(d).

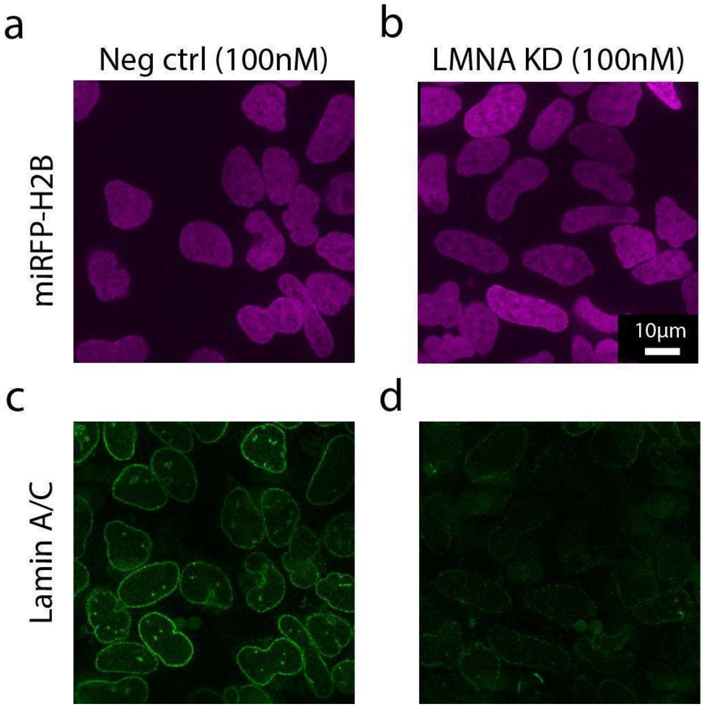

Supplementary Fig. 20. Immunofluorescence staining of U2OS cells with Lamin A/C knockdown (100nM siRNA)

(a) and (b), Confocal fluorescence Images showing miRFP-670 tagged nuclei of cells with 100nM non-targeting siRNA (negative control) and 100nM LMNA siRNA (LMNA KD). The experiment was repeated three times independently with similar results.
(c) and (d), Confocal fluorescence images showing the Immunofluorescence staining of Lamin A/C in cells with 100nM non-targeting siRNA (negative control) and 100nM LMNA siRNA (LMNA KD). The experiment was repeated three times independently with similar results.

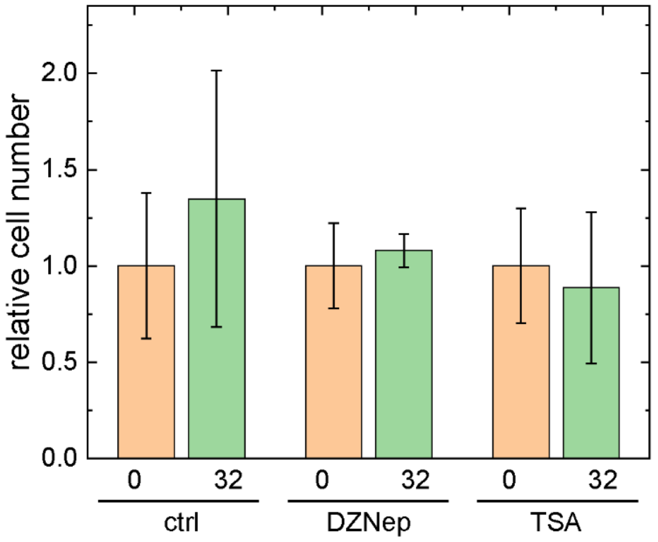

Supplementary Fig. 21. The relative number of U2OS cells after 32 hours of drug treatment
Quantification of the relative cell number of wild-type cells with no drug, DZNep, and TSA treatment; cell numbers are normalized to those at day 0. Cells were treated for 32 hours. Data are averaged from two biological replicates.

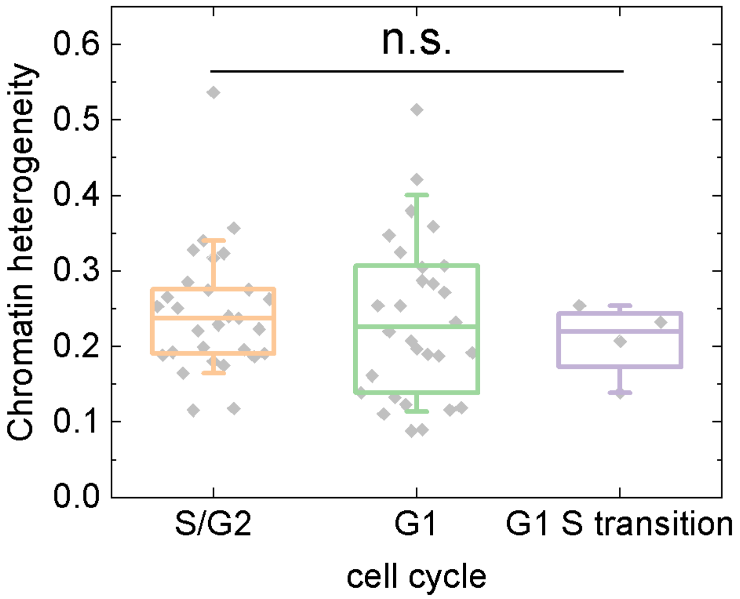

Supplementary Fig. 22. Chromatin heterogeneity at different cell cycles of U2OS cells
Quantification of chromatin heterogeneity in different cell cycles of U2OS cells, including S/G2 (n=), G1 (n=), G1 S transition (n=). n.s., no statistical significance is found according to one-way ANOVA statistical tests of 3 groups. Data are from two biological replicates.

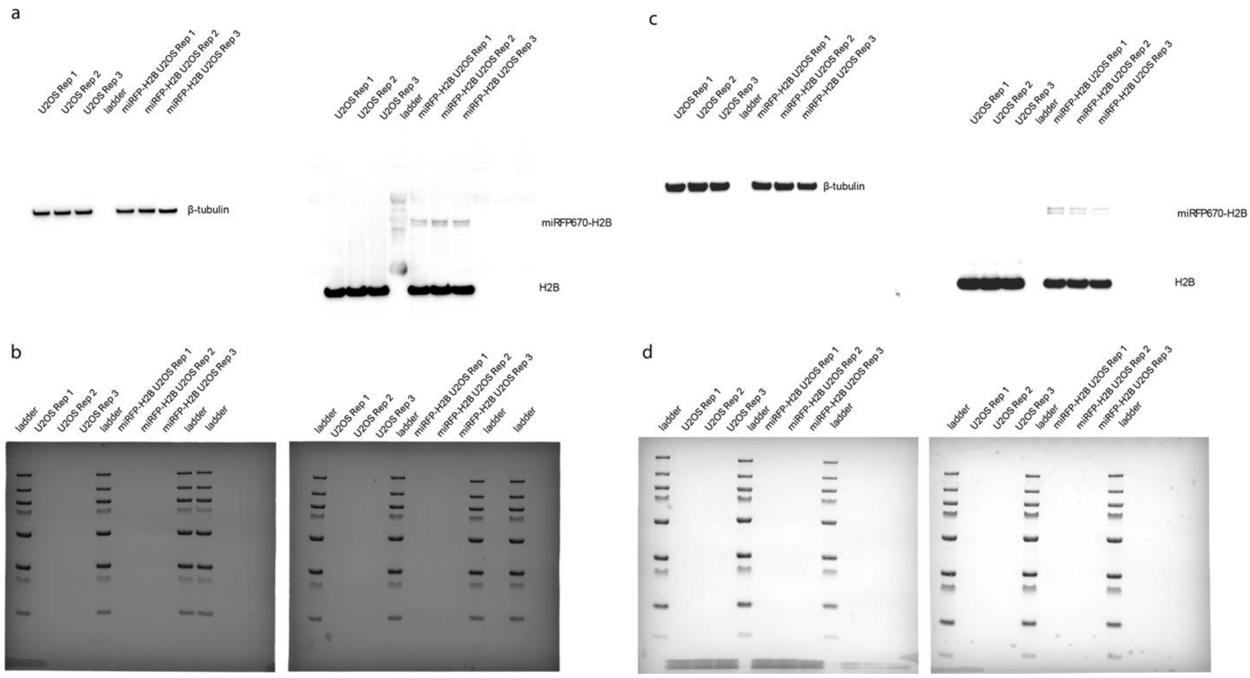

Supplementary Fig. 23. Uncropped Western blot images of U2OS and HeLa cells
(a), Uncropped Western blot of wild-type and miRFP670-H2B U2OS cells, stained with anti-H2B antibody. β-Tubulin is used as loading control and stained with anti- β-tubulin antibody.
(b), Uncropped images of the molecular weight ladder corresponding to (a).
(c), Uncropped Western blot of wild-type and miRFP670-H2B HeLa cells, stained with anti-H2B antibody. β-Tubulin is used as loading control and stained with anti- β-tubulin antibody.
(d), Uncropped images of the molecular weight ladder corresponding to (c).

Supplementary Movie

Supplementary Movie 1. A representative video showing the growth of 'Corelet' condensates in wild-type U2OS cells upon blue light activation.
