# Supplementary material for: Chromatin heterogeneity modulates nuclear condensate dynamics and phase behavior
Source: Nat Commun. 2025 Jul 11;16:6406. doi: 10.1038/s41467-025-60771-9 (PMC12254321; doi:10.1038/s41467-025-60771-9)
Supplement: Supplementary file 3 — Description of Additional Supplementary Files [file 41467_2025_60771_MOESM3_ESM.pdf]

Description of Additional Supplementary Files

File Name: Supplementary Movie 1
Description: A representative video showing the growth of 'Corelet' condensates in wild-type U2OS cells upon blue light activation.
